# The Relationship of Metabolic Syndrome with Stress, Coronary Heart Disease and Pulmonary Function - An Occupational Cohort-Based Study

**DOI:** 10.1371/journal.pone.0133750

**Published:** 2015-08-14

**Authors:** Miroslaw Janczura, Grazyna Bochenek, Roman Nowobilski, Jerzy Dropinski, Katarzyna Kotula-Horowitz, Bartosz Laskowicz, Andrzej Stanisz, Jacek Lelakowski, Teresa Domagala

**Affiliations:** 1 Department of Anesthesiology and Intensive Pulmonary Care, The John Paul II Hospital, Krakow, Poland; 2 Department of Internal Medicine, Jagiellonian University School of Medicine, Krakow, Poland; 3 Unit of Rehabilitation in Internal Diseases, Faculty of Public Health, Jagiellonian University School of Medicine, Krakow, Poland; 4 Department of Radiology and Diagnostic Imaging, The John Paul II Hospital, Krakow, Poland; 5 Department of Bioinformatic and Telemedicine, Jagiellonian University School of Medicine, Krakow, Poland; 6 Department of Electrocardiology, Institute of Cardiology, The John Paul II Hospital, Krakow, Poland; Medical University Hamburg, University Heart Center, GERMANY

## Abstract

**Background and Aims:**

Higher levels of stress impact the prevalence of metabolic syndrome (MetS) and coronary heart disease. The association between MetS, impaired pulmonary function and low level of physical activity is still pending assessment in the subjects exposed to stress. The study aimed to examine whether higher levels of stress might be related to MetS and the plaque presence, as well as whether MetS might affect pulmonary function.

**Design and Methods:**

The study embraced 235 police officers (mean age 40.97 years) from the south of Poland. The anthropometrics and biochemical variables were measured; MetS was diagnosed using the International Diabetes Federation criteria. Computed tomography coronary angiography of coronary arteries, exercise ECG, measurements of brachial flow-mediated dilation, and carotid artery intima-media thickness were completed. In order to measure the self-perception of stress, 10-item Perceived Stress Scale (PSS-10) was applied. Pulmonary function and physical activity levels were also addressed. Multivariate logistic regression analyses were applied to determine the relationships between: 1/ incidence of coronary plaque and MetS per se, MetS components and the number of classical cardiovascular risk factors, 2/ perceived stress and MetS, 3/ MetS and pulmonary function parameters.

**Results:**

Coronary artery atherosclerosis was less associated with MetS (OR = 2.62, 95%CI 1.24–5.52; p = 0.011) than with a co-existence of classical cardiovascular risk factors (OR = 5.67, 95% CI 1.07–29.85, p = 0.03; for 3 risk factors and OR = 9.05; 95% CI 1.24–66.23, p = 0.02; for 6 risk factors, respectively). Perceived stress increased MetS prevalence (OR = 1.07, 95% CI 1.03–1.13; p = 0.03), and impacted coronary plaque prevalence (OR = 1.05, 95% CI 1.001–1.10; p = 0.04). Leisure-time physical activity reduced the chances of developing MetS (OR = 0.98 95% CI 0.96–0.99; p = 0.02). MetS subjects had significantly lower values of certain pulmonary function parameters.

**Conclusions:**

Exposure to job-specific stress among police officers increased the prevalence of MetS and impacted coronary plaque presence. MetS subjects had worse pulmonary function parameters. Early-stage, comprehensive therapeutic intervention may reduce overall risk of cardiovascular events and prevent pulmonary function impairment in this specific occupational population.

## Introduction

Cardiovascular diseases (CVD) account for a substantial amount of deaths worldwide, also in Poland. In the developed countries metabolic syndrome (MetS) affects up to 25% of the population and continues to spread, becoming a major clinical and public health problem, mainly due to its association with CVD [1]. In 2005, the American Diabetes Association and the European Association for the Study of Diabetes emphasized the need to identify CVD risk associated with MetS [2]. Meta-analyses of prospective studies revealed the risk of cardiovascular morbidity and mortality to be 2-fold higher in the patients with MetS [3,4]. There is an on-going debate about viability of MetS as a predictor of CVD risk, and whether it might be a more effective predictor than individual risk factors.

The relationship between individual lifestyle, in particular smoking, psychosocial or work stress, and lack of regular physical activity with morbidity and mortality due to CVDs was demonstrated [5–10]. Physical inactivity is a health behavior strongly associated with obesity and MetS [11]. Despite popular assertion that lack of physical activity is harmful to health, a large proportion of the world’s population remains physically inactive. The lack of regular physical activity or physical inactivity is responsible for 6–10% of major non-communicable diseases, whereas individual life expectancy may increase in the subjects with higher physical activity level [12].

In clinical practice the risk of atherosclerosis is established through the identification of key risk factors for CVD. The assessment of endothelial function (flow-mediated dilation; FMD) and the measurement of carotid artery intima-media thickness (IMT) can also be helpful in assessing the advancement of atherosclerosis and prediction of future CVD outcomes [13,14].

The most commonly used, non-invasive techniques for assessing the severity of atherosclerosis consist in the calcium score and computed tomography coronary angiography (CTCA). Despite many controversies regarding the diagnostic efficacy of CTCA, some reports praise its appreciable potential for assessing the presence of coronary plaque and, to some extent the severity of atherosclerosis in MetS subjects [15,16]. Several studies demonstrated a clear linkage between the work-related stress and the key risk factors for CVD [17,18]. The very nature of police work imposes a significant psychological burden on the police officers. Only a few studies had investigated this occupational group, yet none of them ever attempted to offer a broad assessment of atherosclerotic coronary plaque presence with the aid of CTCA [17,19,20].

It is for this reason that an integrated prevention strategy, based on a systematic evaluation of the total risk of a disease at an individual level, appears vital for this particular occupational group. It is well expected to facilitate reaching the subjects earlier in the course of a vascular disease, and possibly also mitigate the attendant risk factors, help reduce clinical manifestation of CVD, as well as other consequences of obesity and MetS (e.g. impairment of pulmonary function).

It was demonstrated that the subjects with pulmonary function impairment had a higher risk of MetS and its individual components than the subjects with a normal pulmonary function [21]. A positive independent relationship between a pulmonary function impairment and abdominal obesity was established [22]. Forced expiratory volume in 1 second (FEV_1_) was regarded as an independent predictor of MetS, although restriction was also related to this syndrome [23–25]. Low pulmonary function was associated with MetS also within a general population [25].

The present cohort study is focused on the police officers, as the individuals particularly exposed to a highly stressful occupation. Therefore many aspects of this study may well be translated into developing an optimal preventive strategy for this particular occupational group. We primarily aimed to investigate whether MetS and its components, as well as CVD risk factors, were in any way correlated with the presence of coronary artery plaque. We also assessed whether stress was related to both MetS and the presence of coronary artery plaque. The relationship between MetS and pulmonary function was also evaluated.

## Materials and Methods

### Study design and population

The present study embraced a cohort of police officers from the southern region of Poland who volunteered for coronary heart disease (CHD) screening. Two hundred and thirty-five consecutive, professionally active subjects (216 men, 19 women), aged 27–58 years, were enrolled.

Physical examination, medical and structured interviews were applied to collect personal and clinical information. Anthropometric measurements, i.e. circumference of waist and hip, height and body weight were also measured. Obesity was defined as BMI>30.0 kg/m^2^ and overweight as BMI >25.0 kg/m^2^. Cardiovascular disease risk was prospectively evaluated through Framingham risk score (FRS).

Subjects were subsequently split into two groups, i.e. either with MetS (n = 109), or without it (n = 126), using the criteria proposed by the International Diabetes Federation: waist ≥94 cm in men and ≥80 cm in women, fasting glucose ≥5.6 mmol/L (100.0 mg/dL) or previously diagnosed type 2 diabetes, hypertriglyceridemia ≥1.7 mmol/L (150.0 mg/dL) or specific treatment for this lipid abnormality, HDL-cholesterol <1.03 mmol/L (40.0 mg/dL) in men and <1.29 mmol/L (50.0 mg/dL) in women, and blood pressure systolic/diastolic ≥130/≥85 mm Hg or treatment of previously diagnosed hypertension [26,27]. The study was approved by the local Ethics Review Committee of the Jagiellonian University, and an informed written consent was granted by all participants.

### Biochemical measurements

Blood samples were taken after a 12-hour overnight fast. Total cholesterol and triglycerides were determined by an enzymatic method, while LDL cholesterol was calculated with the Friedewald formula. Basic biochemical tests (liver enzymes, glucose, total protein, urea, uric acid and creatinine) were carried out with the aid of Vitros 350 biochemical analyser. Ultrasensitive CRP in the serum was determined by nephelometry and tissue necrotic factor-α (TNF-α) in the plasma by ELISA.

### Treadmill exercise ECG testing

Symptom-limited maximal treadmill exercise tests were performed using the standard Bruce protocol [28] on a treadmill (Marqueette Electronics). The end point of the test was usually fatigue, or an individual inability to keep pace with the treadmill, unless another indication for test termination was met first. A 12-lead ECG was obtained every minute during the exercise, at peak exertion, and in the recovery phase. Exercise workload was estimated in metabolic equivalents (METs), where 1 MET = 3.5 ml/kg per minute of oxygen consumption. Positive test result was determined when ST-segment depressed or elevated horizontally >0.1 mV 80 ms after the J point (ST80) from normal baseline, in three consecutive beats. Criteria for ischemic response also included slow upsloping ST-segment depression >0.15 mV with ST-segment slope >1.0 mV/sec, and downsloping ST-segment depression occurred when the depression was 0.1 mV, and the ST-segment slope was -1.0 mV/sec.

### Ultrasound imaging

The measurements of brachial artery diastolic response (flow-mediated dilation; FMD) and the average thickness of the carotid intima-media thickness (IMT) were obtained using an ultrasonograph (Sequoia 512, Mountain View, Ca, USA) with a 6 MHz linear transducer.

The measurements of endothelium-dependent FMD of brachial artery in response to reactive hyperemia were evaluated non-invasively, in compliance with the ultrasound method described by Celermajer et al. [29]. All measurements were taken on the right brachial artery 2–3 centimeters above antecubital fossa after a patient had stayed in the supine position for 5 min. Reactive hyperemia was induced by the inflation of sphygmomanometer cuff around the forearm to 200 mmHg for 5 min. Endothelium-dependent response was construed as the dilation of the brachial artery induced by an increased flow. The subjects were studied in a fasting state (between 7.00 p.m. and 8.00 a.m.); exposure to caffeine, smoking and exercise were prohibited prior to the imaging study. All FMD measurements were performed on the same apparatus, by the same person, and repeated in a period of 1–2 months. IMT measurements of the distal wall of the carotid artery were taken in three locations: 1. common carotid artery (2 cm below the bulb), 2. carotid artery bulb, and 3. proximal internal carotid artery. The final IMT value was the mean from all measurements on both carotid arteries.

### Computed tomography coronary angiography

Out of the entire cohort, 154 subjects (65.53%) underwent computed tomography coronary angiography (CTCA). All scans were performed by dual source CT scanner (Somatom Definition; Siemens Medical Solutions). Coronary Computed tomography was performed by a 64-slice configured CT. Data were acquired in a craniocaudal direction with a detector collimation 2x32x0.6 mm and a gantry rotation time of 0.33 seconds. Image acquisition was performed during an inspiratory breath hold of ca. 10 s. Image reconstruction was retrospectively gated to the ECG. CT image was reconstructed by mono-segmental mode, using the section thickness of 0.6 mm and a smooth-tissue convolution kernel (B26F). All images were evaluated using a remote workstation with dedicated software (Siemens Leonardo Station). The contrast material was administrated in an antecubital vein in the amount of 70–100 ml, at the rate of 5.5 mL/s [30]. The observer then compared the minimal lumen area to an arterial one at an appropriate reference site, in a non-diseased arterial segment, in the closest proximity to the lesion, preferably with no branch vessels in between. The subjects with supraventricular and ventricular arrythmias, renal insufficiency, and confirmed allergy to contrast media were excluded.

### Physical activity

Physical activity was evaluated using the International Physical Activity Questionnaire-Long Form (IPAQ-LF). The items in the IPAQ-LF form were structured to provide separate domain specific scores for walking, moderate-intensity and vigorous-intensity activity within each of the work, transportation, domestic chores and gardening (yard) and leisure-time domains. Domain-specific scores required summation of the scores for walking, moderate-intensity and vigorous-intensity activities within the specific domain, whereas activity-specific scores required summation of the scores for the specific type of activity across domains.

Based on the questionnaire data, weekly physical activity score was calculated. Using the Ainsworth et al. [31] Compendium an average Metabolic Equivalent of Task (MET) score was derived for each type of activity. A specific method was also used with a view to correcting MET values, so as to account for personal variation in sex, body mass, height, and age, to provide more accurate estimates of individual physical activity level [32]. The resulting MET value was referred to as a “corrected MET” value, and expressed as the corrected MET-minutes/kg of body weight.

### Perceived Stress Scale

In order to measure the self-perception of stress Perceived Stress Scale-10 (PSS-10) in Polish adaptation was applied [33,34]. It is a measure of the extent to which situations in one’s life are perceived as stressful. Specifically structured questions were designed to probe how unpredictable, uncontrollable, and overloaded respondents find their lives. Participants are asked to respond to each question on a 5-point Likert scale ranging from 0 (never) to 4 (very often), indicating how often they have felt or thought in a certain way within the past month. Overall score was completed through the reverse scoring of the four positively-worded items and summing all item scores. Scale scores ranged 0–40, with the higher scores indicating higher levels of stress.

Cronbach's alfa was used to evaluate reliability, while exploratory and confirmatory factor analyses were applied to evaluate validity of PSS-10. To confirm the observations and fit the two-factor model, a confirmatory factor analysis was performed. The goodness of fit of models were assessed using Goodness of Fit Index (GFI) and the Root Mean Square Error of Approximation (RMSEA). The following statistics were obtained: GFI = 0.916 and RMSEA = 0.077. The PSS-10 demonstrated good reliability, as Cronbach's alpha was 0.85 (0.84–0.86).

### Pulmonary function

Standard spirometry was performed (Master Screen MS PFT, Jaeger, Wurzburg, Germany) according to the 2005 American Thoracic Society/European Respiratory Society recommendations [35]. The best one of the three repeatable manoeuvres was recorded. The measured volumes were adjusted for sex, age, and height using equations from a reference population of non-smoking Caucasians, and were expressed as a percentage of the predicted values. The following values were selected for the study: forced expiratory volume in 1 second (FEV_1_), forced vital capacity (FVC), forced expiratory flow (FEF_25_, FEF_50_, FEF_75_), vital capacity (VC), expiratory reserve volume (ERV), FEV1 to FVC ratio (FEV_1_%FVC), and FEV1 to VC ratio (FEV_1_%VC). Calibration check was performed every morning by using a 3-liter syringe. In order to verify that the spirometer remained within the desired calibration limits (±3%), the maneuvers were repeated 6–8 times, if required.

### Statistical analyses

Statistical analysis was completed using *STATISTICA 10 PL* and IBM SPSS Statistics 21. For comparison of the two groups a nonparametric Mann-Whitney test was used. In order to evaluate the relationship between respective qualitative variables contingency tables were created and the value of the chi-square (χ2) was calculated. Coronary plaque, and MetS were treated as binary variables. To evaluate the relationships between coronary plaque, MetS and its components, CVD risk factors, and perceived stress score, as well as MetS and stress, separate univariate logistic regression models were applied.

Multivariate logistic regression models were constructed to assess the respective relations of different variables for the incidence of coronary plaque, i.e. a number of CVD risk factors without, and after adjustment for perceived stress score; MetS per se (one-dimensional model), and after adjusting for age, sex, smoking, and perceived stress score, and also the impact of MetS components after adjusting for age, sex, smoking and perceived stress score. To obtain crude and adjusted odds ratios (ORs) and 95% confidence intervals (CIs) the models of multivariate logistic regression were used. In order to identify the pulmonary function affecting factors, stepwise multiple regression models were devised.

As independent variable applied MetS components. For evaluating the correlations between respective variables, Spearman's rank correlation coefficient was used. P<0.05 indicated statistical significance.

## Results

### Establishing the extent of coronary atherosclerosis and clinical characteristics

Clinical characteristics of the participants (216 M, 19 F) are comprised in Table 1, while characteristics of the study subjects stratified by MetS status are presented in Table 2. The MetS subjects, as compared to the non- MetS subjects, showed higher (although not significantly; p = 0.053) prevalence of stable CHD, diagnosed by typical clinical coronary symptoms and positive exercise ECG testing (Table 2). Among 194 (82.55%) subjects (88 with and 106 without MetS) who underwent exercise stress testing, positive result was significantly more frequent in the MetS subjects (p = 0.012), whereas exercise workload was markedly lower in this group [12.28 (3.83) METs vs. 13.45 (2.70) METs, p = 0.019].

**Table 1:** Clinical characteristics of the study subjects.

Study variable	Value
N (F)	235 (19)
Age, years*	40.97 (6.26)
Framingham Risk Score*	5.82 (5.11)
W/H ratio*	0.94 (0.06)
Overweight, n (%)	101 (42.97)
Obesity, n (%)	103 (43.83)
Dyslipidemia, n (%)	120 (51.06)
Diabetes mellitus, n (%)	7 (2.97)
Hypertension, n (%)	104 (44.25)
Stable CHD, n (%)	17 (7.23)
Positive family history of CHD, n (%)	128 (54.46)
History of myocardial infarction, n (%)	1 (0.42)
PCI, n (%)	5 (2.13)
Smoking status, n (%) Never Current Past Pack-years*	96 (40.85)79 (33.62)60 (25.53)17.68 (11.83)
COPD, n (%)	0
Treatments, n (%) Aspirin ACEI/ARB β-blocker Ca-blocker Diuretics Lipid-lowering drugs Anti diabetic drugs	8 (3.40)44 (18.72)42 (17.87)14 (5.97)30 (12.76)13 (5.53)7 (2.97)

* Data are expressed as mean (SD); Framingham Risk Score, 10-year risk of developing coronary heart disease; W/H ratio, waist/hip; CHD, coronary heart disease; PCI, percutaneous coronary intervention; BMI, body mass index; ACEI, Angiotensin converting enzyme inhibitors; ARB, Angiotensin II receptor blockers; COPD, chronic obstructive pulmonary disease. Obesity was defined as BMI>30.0 kg/m^2^ and overweight as BMI >25.0 kg/m^2^.

**Table 2:** Characteristics of the study subjects stratified by metabolic syndrome status.

Study variable	Metabolic Syndrome	Non-Metabolic Syndrome	p value
N (F)	109 (4)	126 (15)	0.038
Age, years	42.54 (6.75)	39.61 (5.48)	<0.001
Framingham Risk Score	8.32 (5.26)	3.43 (2.99)	<0.0001
BMI, kg/m^2^	32.27 (3.73)	27.78 (3.28)	<0.0001
W/H ratio	0.98 (0.04)	0.91(0.06)	<0.0001
Systolic blood pressure, mm Hg	137.93 (16.93)	124.15 (15.53)	<0.0001
Diastolic blood pressure, mm Hg	87.66 (10.48)	80.36 (9.79)	<0.0001
Stable CHD, n (%)	12 (11.01)	5 (3.96)	0.053
Positive result of stress ECG; n (%)*	18 (18.37)	7 (6.60)	0.012
Coronary plaque burden, n (%)**	29 (37.66)	14 (18.18)	0.007
FMD, %	7.80 (3.23)	8.17 (3.71)	0.59
IMT, mm	0.59 (0.10)	0.54 (0.09)	0.0002
Smoking status, n (%)			
Never Current Past	35 (32.11)40 (36.69)34 (31.19)	61 (48.41)39 (30.95)26 (20.63)	0.010.350.06
Total cholesterol, mmol/L	5.61 (1.01)	5.02 (0.88)	<0.0001
LDL cholesterol, mmol/L	3.25 (0.86)	3.02 (0.76)	0.056
HDL cholesterol, mmol/L	1.12 (0.25)	1.37 (0.35)	<0.0001
Triglycerides, mmol/L	2.92 (1.76)	1.35 (0.75)	<0.0001
Glucose, mmol/L	5.72 (1.29)	5.08 (0.39)	<0.0001
CRP, mg/L	2.18 (2.35)	1.55 (1.78)	0.0001
TNF-α, pg/mL	1.70 (2.85)	1.25 (2.04)	0.06

Data are expressed as mean (SD); BMI, body mass index; W/H, waist/hip; CHD, coronary heart disease; FMD, flow-mediated dilation; IMT, intima-media thickness; LDL, low density lipoprotein; HDL, high density lipoprotein; CRP, C-reactive protein; TNF-α, tissue necrotic factor-α;

* n (%) subjects who underwent stress ECG;

** n (%) subjects who underwent computed tomography coronary angiography.

CTCA was completed in 154 (65.53%) subjects (MetS n = 77, non-MetS n = 77). Coronary artery plaques were found in 43 (27.92%) of study subjects (non-calcified in 15, calcified in 23, and mixed in 5). Amongst 17 CHD subjects only in 11 of them coronary artery burden was revealed by CTCA. Out of the remaining 137 non-CHD subjects, CTCA showed significant atherosclerotic plaques and/or varying vascular stenosis ranging 40%-70% in 35 subjects (32.30% MetS and 15.27% non-MetS), despite no clinical symptoms of atherosclerosis, and a negative outcome of stress ECG testing.

### Metabolic syndrome, cardiovascular risk factors, coronary atherosclerosis, carotid IMT and FMD

As shown in CTCA, the MetS subjects had a higher prevalence of any type of coronary artery atherosclerosis (coronary plaque and/or stenosis), as compared to the non-MetS subjects (p<0.007). Logistic regression demonstrated that MetS (binary variable) significantly increased by 2.5-fold the chance of coronary artery atherosclerosis (OR = 2.62, 95%CI 1.24–5.52, p = 0.01; Table 3). Age and cigarette smoking were the variables that affected the relationship (models 2,3,5). After adjustment for age, we found OR = 2.16 (95% CI 1.02–4.78; p = 0.04), and after further adjustment for smoking the relationship appeared even weaker (OR = 2.09, 95% CI 1.02–4.76; p = 0.04). Out of all MetS components only hypertension impacted the incidence of coronary plaque in the univariate model (OR = 1.03, 95% CI 1.009–1.054; p = 0.004; OR = 1.049, 95% CI 1.01–1,08; p = 0.007, for systolic and diastolic blood pressure, respectively) as well as in the multivariate analysis (Table 4).

**Table 3:** Models of logistic regression analysis: assessment of the impact of metabolic syndrome on the coronary plaque prevalence, depending on the correcting variable.

Regression Model	Estimate	95% CI	Wald Chi-Square	P	OR	95% CI
0*	0.053	-0.024–0.131	1.87	0.17	1.05	0.97–1.14
1	0.96	0.21–1.70	6.51	0.01	2.62	1.24–5.52
2	0.76	-0.02–1.56	3.64	0.0451	2.16	1.02–4.78
3	0.73	0.25–1.36	3.53	0.0494	2.09	1.02–4.76
4	0.94	0.05–1.83	4.38	0.030	2.56	1.05–6.26
5	0.71	-0.21–1.64	2.34	0.0498	2.04	1.01–5.17

Data are expressed as ORs and corresponding 95% CIs.

OR, odd ratio; CI, confidence interval;

Model 0*—perceived stress score, unadjusted;

Model 1 –metabolic syndrome, unadjusted,

Models 2–5, metabolic syndrome adjusted for: 2—age; 3—age, sex and smoking;

4—sex, smoking and perceived stress score; 5—age, sex, smoking and perceived stress score.

**Table 4:** Results of logistic regression analysis: odds ratios for coronary plaque prevalence according to MetS components adjusted for age, sex, smoking and perceived stress score.

Variable	Estimate	95% CI	Wald Chi-Square	P	OR	95% CI
WaistCircumference*	0.035	-0.010–0.081	2.303	0.129	1.035	0.98–1.08
Hypertension	0.047	0.011–0.083	6.650	0.009	1.048	1.011–1.087
Hypertriglycerydemia	0.047	-0.353–0.448	0.055	0.814	1.048	0.702–1.565
Low HDL cholesterol	0.103	-0.252–0.458	0.327	0.567	1.108	0.776–1.581
Elevated FBG	0.160	-0.217–0.539	0.706	0.40	1.17	0.804–1.715
Age	0.103	0.027–0.180	7.321	0.006	1.109	1.028–1.197
Sex	0.912	-0.783–2.608	1.132	0.287	2.491	0.456–13.583
Smoking	0.468	-0.403–1.339	1.129	0.287	1.597	0.668–3.817
PSS	0.051	0.001–0.098	4.149	0.041	1.051	1.001–1.103

Data are expressed as ORs and corresponding 95% CIs.

OR, odd ratio; CI, confidence interval; PSS, perceived stress score.

* ≥ 94/80 cm (men/women). Hypertension was defined as blood pressure systolic/diastolic ≥130/≥85 mm Hg or treatment of previously diagnosed hypertension; hypertriglyceridemia was defined as total triglycerides ≥1.7 mmol/L (150.0 mg/dL) or specific treatment for this lipid abnormality, low HDL-cholesterol was defined when cholesterol in this lipoprotein fraction was <1.03 mmol/L (40.0 mg/dL) in men and <1.29 mmol/L (50.0 mg/dL) in women; elevated fasting blood glucose (FBG) was defined when fasting glucose ≥5.6 mmol/L (100.0 mg/dL) or previously diagnosed type 2 diabetes.

The odds ratios for coronary plaque presence, relative to a number of CVD risk factors, are shown in Table 5. A trend towards an increased prevalence of coronary atherosclerotic plaques with an increasing number of CVD risk factors (i.e. obesity, dyslipidemia, hypertension, dysglicaemia, cigarette smoking and CV history) was established. Each additional consecutive risk factor (above two) increased the OR for the atherosclerotic coronary plaque presence. In multivariate model, adjusted for age, sex and stress, each successively added risk factor had greater odds for coronary atherosclerosis (OR = 5.67, 95% CI 1.07–29.85, p = 0.03; for 3 risk factors and OR = 9.05; 95% CI 1.24–66.23, p = 0.02 for 6 risk factors), when compared to the subjects with 0–2 CVD risk factors (Table 5).

**Table 5:** Results of logistic regression analysis: odds ratios of plaque prevalence according to CVD risk factors.

Number of CVD risk factors	Model 1			Model 2		
	OR	95% CI	P	OR	95% CI	P
3	7.15	1.47–34.76	0.01	5.67	1.07–29.85	0.03
4	8.83	1.83–42.56	0.006	6.85	1.34–34.93	0.01
5	10.60	1.99–56.25	0.005	6.50	1.060–39.90	0.04
6	11.00	1.60–75.25	0.013	9.05	1.24–66.23	0.02

Data are expressed as ORs and corresponding 95% CIs,

OR, odd ratio; CI, confidence interval; CVD, cardiovascular disease;

Reference point for CVD risk factors is 0–2.

Model 1- Adjusted for age and sex;

model 2—Adjusted for age, sex and perceived stress score.

Apart from the MetS components, a significantly higher carotid IMT value, plasma CRP, but no TNFα levels, and brachial artery FMD in the MetS subjects were found (Table 2). The mean carotid IMT was significantly higher in the subjects with coronary plaque presence, as compared to the subjects without it (0.61±0.13 mm vs. 0.56±0.10 mm; p = 0.02). The highest mean IMT value was found in the MetS subjects with coronary plaque, as compared to the non-MetS subjects, and the ones without any coronary plaque presence (p = 0.006).

Carotid IMT value positively correlated with a number of CVD risk factors, all MetS components, plasma CRP levels, and inversely correlated with brachial FMD (S1 Table). The mean baseline diameter of brachial artery was significantly higher in the MetS subjects and inversely correlated with FMD value. The dilatory response of brachial artery was associated with a single MetS component only (waist circumference).

### Stress, metabolic syndrome, coronary atherosclerosis, and carotid IMT

The mean score of perceived stress was 16.66 (scores range: 0–40; 41.65%). It was significantly higher in the MetS subjects (17.55 [43.87%] *vs*. 15.78 [39.45%]; p = 0.03). In univariate analysis perceived stress increased the chance of MetS prevalence (OR = 1.07, 95% CI 1.03–1.13; p = 0.03). The level of stress correlated with three MetS components, i.e. waist circumference (r = 0.17, p = 0.03), triglycerides plasma levels (r = 0.19, p = 0.002) and blood pressure (r = 0.19, p = 0.01; r = 0.17, p = 0.03; for systolic and diastolic, respectively; S1 Table).

The association between plaque prevalence and MetS (binary variable), depending on the correcting variables, are presented in Table 3. Perceived stress was not associated with the prevalence of coronary plaque in the univariate model (OR 1.05, 95% CI 0.97–1.14; model 0). In the multivariate logistic regression model, age, gender, cigarette smoking and PSS, were significant (models 2–5). The relationship of all MetS components with the prevalence of coronary plaque, following an adjustment for age, sex, cigarette smoking and perceived stress score, is presented in Table 4. In multivariate logistic regression analysis, stress affected the prevalence of coronary plaque (OR = 1.05, 95% CI 1.001–1.10; p = 0.04), and out of MetS components only hypertension had its effect. Age was also a variable related to the incidence of plaque. In turn, as shown in Table 5, perceived stress appeared the variable that actually modified the effect of more than two CVD risk factors on the incidence of coronary lesions.

The association of perceived stress with the values of carotid artery IMT was weak (r = 0.15, p = 0.052), and altogether non-existent for FMD. No association whatsoever was found between the perceived stress score and the level of individual physical activity. The nature of relationships between select study variables are presented in S1 Table.

### Physical activity and metabolic syndrome

Out of all physical activity domains only leisure-time physical activity was significantly lower in the MetS subjects (p = 0.0001; Table 6). Logistic regression showed that leisure-time physical activity reduced the chances of developing MetS (OR = 0.98, 95% CI 0.96–0.99; p = 0.022). On the other hand, the intensity of physical activity associated with transportation and total walking was not significantly lower in the MetS subjects (p = 0.08). There were no differences between the respective study groups with regard to moderate, vigorous and total physical activity (Table 6).

**Table 6:** Spirometry test results and the intensity of physical activity in the study subjects stratified by metabolic syndrome status.

Study variable	Metabolic Syndromen = 109 (F; 4)	Non-Metabolic Syndrome n = 126 (F; 15)	p value
Spirometry test			
FEV_1_	99.77 (10.36)	103.55 (14.79)	0.01
VC	100.54 (9.34)	105.31 (14.45)	0.0002
FVC	101.55 (9.73)	106.42 (14.06)	0.0004
FEV1%VC	81.04 (7.70)	80.99 (9.18)	0.65
FEV1%FVC	80.36 (6.06)	80.97 (8.07)	0.73
FEF25	99.42 (19.49)	102.64 (18.61)	0.31
FEF50	89.73 (25.09)	89.17 (22.23)	0.98
FEF75	61.87 (22.30)	65.85 (24.41)	0.41
ERV	56.03 (33.34)	78.48 (47.57)	0.001
Physical activity (Corrected MET-minutes/week/kg of body weight)			
Work-related	69.61 (69.18)	73.64 (74.68)	0.65
Transport-related	32.37 (38.96)	44.38 (52.16)	0.08
Domestic and gardening activities	48.02 (52.47)	44.91 (57.00)	0.48
Leisure time	18.32 (25.84)	29.67 (27.15)	0.0001
Total walking	58.45 (57.34)	77.82 (73.88)	0.08
Total moderate	58.11 (62.49)	65.03 (69.65)	0.33
Total vigorous	52.99 (55.33)	52.01 (59.01)	0.68
Total physical activity	168.32 (117.62)	192.60 (151.84)	0.22

Data (percent predicted) are expressed as mean (SD); FEV1, forced expiratory volume in 1 second; VC, Vital Capacity; FVC, forced vital capacity; FEV_1_%VC, Tiffenau index; FEV1/FVC, a ratio of forced expiratory volume in 1 second (FEV1) to a forced vital capacity (FVC); FEF, forced expiratory flow; ERV, expiratory reserve volume; MET, Metabolic Equivalent of Task.

### Pulmonary function and metabolic syndrome

The MetS subjects had significantly lower values of FEV_1_, FVC and VC than the non-MetS subjects (Table 6). However, the values in both groups remained within normal limits, though. As regards the individual values of pulmonary function parameters, only 2 out of 235 subjects (0.85%) had FEV_1_, VC and FVC under the 5^th^ percentile, and only 7 subjects (3%) had FEV_1_/FVC ratio under the 5^th^ percentile. FEV_1_%FVC and FEV_1_%VC ratios did not differ between the groups. Only ERV was evidently diminished, and MetS subjects had significantly lower ERV than the non-MetS subjects (p = 0.001). Neither of the groups differed with respect to FEFs. The plasma levels of CRP negatively correlated with FEV_1_ (r = -0.16, p = 0.03), ERV (r = -21, p = 0.006), FVC (r = -0.20, p = 0.008), and FEF_75_ (r = -0.21, p = 0.005). There were no associations between TNF-α and pulmonary function parameters.

Out of all MetS components, waist circumference negatively correlated with FEV_1_ (r = -0.18, p = 0.01), VC (r = -0.24, p = 0.0007), FVC (r = -0.26, p = 0.0003), and ERV (r = -0.34, p = <0.0001). Also BMI negatively correlated with VC (r = -0.17, p = 0.01), FVC (r = -0.20, p = 0.004) and ERV (r = -0.36, p = 0.0001). All relationships between respective variables are shown in S2 Table. In the stepwise analysis, waist circumference significantly influenced FEV_1_ (β = -0.17, p = 0.04), ERV (β = -1.34, p<0.0001), FVC (β = -0.24, p = 0.0004), whereas plasma triglycerides levels significantly affected VC (β = -2.13, p = 0.01).

## Discussion

This study is the first to address the cardiovascular risk profiles, including coronary plaque presence and surrogate markers of atherosclerosis in a cohort of police officers. We also assessed the relationship of perceived stress with MetS prevalence, and the coronary atherosclerosis. Since the interrelationship of CVD with the pulmonary function and MetS has recently come to some prominence amongst the investigators, pertinent spirometry variables were also assessed.

### Metabolic syndrome, cardiovascular risk factors, and coronary atherosclerosis

High incidence of risk factors for CVD was established in the study group, especially obesity affected 43.83% of the study subjects, while MetS—46%. Those proportions greatly exceeded the incidence of those factors within general Polish population within the same age range [36,37]. Frequently, the incidence of obesity and overweight among policemen was also highlighted by other investigators [17,38]. In comparison to general population, police officers are up to 1.7 times more likely to develop CVD [39,40].

Motillo et al. in their 2010 meta-analysis demonstrated that MetS was associated with a 2-fold increase in risk for morbidity and mortality CVD, and a 1.5-fold increase in the risk for all-cause mortality [3]. Even though deemed a useful, though controversial construct, for many years there has been no consensus as to MetS significance in clinical practice [41,42]. Some years back, it attracted substantial criticism from the American Diabetes Association for its modest consistency and rather limited clinical application [43].

In 2009, a consensus statement on the definition of the MetS, representing the views of six major organizations and societies, was published [27]. In this document they underscored the need to identify the cardiovascular risk associated with MetS. In contrast to previous clinical definitions, which have differed in the priority given to obesity, the waist measurement would to be a useful preliminary screening tool, while on a temporary basis the national or regional cut-points for waist circumference may well be used. Obesity plays a central role in the development of MetS, and should therefore be considered a key component of any clinical definitions. Especially obesity in isolation, before the actual hallmarks of metabolic dysfunction that typify MetS have developed.

Clinical definitions of MetS were designed to identify a population at high lifetime CVD and type 2 diabetes risk, but in the absence of several major risk factors (age, gender, cigarette smoking, total and LDL cholesterol, CV history), are not optimal risk prediction devices for either. The over 33-year follow-up study demonstrated MetS to be a risk factor independent of other established CVD risk factors, indicating the longer-term prognostic value of MetS for CVD, over and above that achieved by short-term global risk calculators [44]. Its presence or absence should therefore be considered an indicator of long-term risk. On the other hand, the short-term (5–10 years) risk is better calculated using the classical algorithms (Framingham), as they include several major CVD risk factors [45]. Despite this, MetS boasts several properties that make it a useful construct, in conjunction with short-term risk prediction algorithms e.g. FRS, and sound clinical judgment for the identification of those at high lifetime risk of CVD and diabetes.

In our study, logistic regression confirmed that each additional CVD risk factor (above 2) increased the chances for the incidence of coronary artery atherosclerotic plaque. Study Wanahita et al. failed to show any evidence of an increased prevalence of coronary artery disease among police officers, as evaluated by calcium scores only, when compared with the general population [46]. We demonstrated that the relationship of MetS alone with coronary atherosclerosis was also of some significance, even though less so than the effect of concomitant, classical CVD risk factors, as detected by CTCA.

Our results remain in agreement with the study of Butler at al., whereas Pigna et al. found that atherosclerotic burden was more strongly correlated with the number of individual MetS-related factors than with the clinical diagnosis of MetS itself [15,16]. On the other hand, Sattar et al. showed that MetS and most of its components were associated with the risk for the newly onset diabetes only in the elderly populations [47]. Some angiographic studies produced inconsistent results with regard to this issue [48–50]. Recently, a prospective, multicenter study provided data that MetS patients were significantly more prevalent, and CHD prognosis was comparable to the patients with 1, but not with 2 MetS components [50]. However, none of the above referenced studies addressed the population of police officers.

### Stress and metabolic syndrome

Significant role of stress in the pathogenesis of MetS and atherosclerotic process has also recently been granted its due acknowledgement, as police work is deemed one of the most stressful professions [38,51,52]. In the present study perceived stress appreciably increased the chance for MetS prevalence. Violanti et al. [51] revealed that three or more of MetS components were encountered in police officers exhibiting the highest levels of posttraumatic stress disorder symptoms, in comparison to the officers allocated to the lowest stress symptom category. The INTERHEART study showed that work stress doubled the risk of CHD, and the data from meta-analysis of the published prospective cohort studies suggested that work stress was associated with 50% excess risk of CHD [8,53]. Accumulated work stress proved a risk factor for CHD, especially among the younger, working-age population and was associated with a higher risk of MetS and incidental obesity [54–56]. As reported by Chandola et al., about 16% of the effect of work stress on CHD may well be attributed to its effect on MetS [57].

Even though PSS-10 scale has been used in various populations, including Poland, very few studies assessed the perceived stress using this scale in police officers [34,58–60]. In our own study the mean PSS-10 scores in MetS and non-MetS subjects were higher than the results demonstrated in Chinese young female police officers (mean 15.2; 38.0%), and were also higher, as compared to the ones yielded by the community residents, as quoted in the original norms [58,61]. Furthermore, in our cohort group the perceived stress value was higher than in the Ramey et al. [38] study where the mean score was 20.0, since the PSS-14 scale was used by the investigators (scores range 0–56; 35.71%). In the recently published study of Carson et al., a correlation between PSS-10 score and BMI was demonstrated [60].

We established that perceived stress level was associated with MetS, and actually correlated with three of its components (waist circumference, triglycerides, and diastolic/systolic blood pressure). We also found that blood pressure and stress was associated with coronary plaques, as well as that stress was the variable that actually modified the effect of more than two CVD risk factors on the incidence of coronary lesions.

There is an increasing evidence of potential role of stress in the pathogenesis of premature CHD, even though the actual mechanisms underlying this association remain unclear [52,53,57,62]. Stress may act alone, or through the development of risk factor clustering, represented by MetS, as well as may impact the other CVD risk factors at different stages of life [54]. In our study, though, perceived stress was not associated with the prevalence of coronary plaque. The long latency period between some distant risk factors and manifest CHD, and the fact that CHD is a multi-etiological disease, make it difficult to distinguish between single causal risk factors [63]. Consequently, the heterogeneity of MetS poses a problem when assessing the risk for CVD, while the actual level of risk has been demonstrated to differ, depending on the combination of its components [64]. Subject to a combination of certain abnormalities, CVD risk may be higher or lower than the estimates for the syndrome considered as a whole.

In our study, the relationship of stress with MetS and its components (hypertension, waist circumference, triglicerydes) was demonstrated. Stress at work may well lead to CHD through direct activation of the neuroendocrine stress pathways, and indirectly through individual health behavior. Higher cortisol level, and hypertension are deemed the adaptive physiological responses to stress [57,65]. In a recently published study, the relationships between shift work, circadian rhythm, and MetS, were consistently documented [66].

In summary, stress may affect the body through direct activation of neuroendocrine responses to stressors, continuing high blood pressure in relation to long-term stress, or more indirectly through bad habits, such as intensified smoking, reduced leisure-time physical activity, and unhealthy diet, which all increase the risk of CHD and the development of obesity and MetS [54,55,57,67–69].

### Metabolic syndrome, carotid IMT and brachial FMD

Localized inflammation in adipose tissue propagates an overall systemic inflammation associated with visceral obesity, insulin resistance and sub-clinical vascular inflammation, which modulates and results in atherosclerotic processes [70]. Sub-clinical atherosclerosis, i.e. increased carotid IMT observed in the MetS subjects, and spontaneous recovery from MetS was associated with a reduced carotid IMT progression [71–73]. The studies addressing the relationship between carotid IMT and CVD risk factors, and MetS components were published by other authors [74,75]. No association between brachial artery FMD and MetS was encountered in the other studies which applied the NCEP-definition of MetS [76,77]. In contrast, in the Framingham Offspring study a significant inverse relationship between the incidence of MetS and FMD was demonstrated [78].

The FMD value depends on the diameter of the artery before occlusion, and no significant differences in the brachial artery diastolic response between respective study groups might result from a larger diameter at baseline in MetS subject. Similar findings were also reported by other investigators [76,78]. The Young Finns Study demonstrated that the increased carotid IMT was associated with a number of CVD risk factors only in the subjects with impaired FMD, although not with those with preserved FMD [79]. The prevalence of established CVD risk factors does not seem to be the sole determinant of endothelial function, as the individuals with normal endothelial function and the patients with various stages of endothelial dysfunction may not, in fact, differ in terms of their respective risk factor profiles [80,81]. Variable endothelial susceptibility of individual subjects to CVD risk factors may well underscore some other, as yet undetermined factors, inclusive of shear stress and genetic predisposition. Considering the age of the study subjects, no significant differences in the mean values of FMD in our study may well be attributed to efficient vasculo-protective mechanisms, despite numerous CVD risk factors.

### Physical activity, CHD and metabolic syndrome

In the present study, intensity of physical activity in both groups was well above the average for adults. Work-related physical activity accounted for about 40% of total physical activity, and those values exceed the corresponding ones published to date [82]. The physical requirements of police work are essential not only with a view to maintaining good health, but also with regard to allowing the individuals to effectively pursue their work duties. We found that leisure-time physical activity reduced the chances of developing MetS. Recent meta-analysis of prospective cohort studies revealed that while a moderate level of work-related physical activity reduced the risk of CHD, high physical activity at work did not actually add to a potential protective effect [9]. On other hand, high level of physical activity at leisure time reduced the risk of CHD within a 20–30% range. In line with the recommendations comprised in the 2008 Physical Activity Guidelines for Americans, “some physical activity is better than none”, and “additional benefits occur with more physical activity” [83].

High total value of physical activity in our study population, and a high profile of relative physical activity (as revealed in the self-assessment questionnaire) gives grounds to believe this might well be due to occupational specifics. Mandatory and formalized occupational activity during working time aggregates with physical activity during leisure time, as is necessary to maintain the occupationally required level of individual physical functionality. Individual pursuit of training regimens in the spare time, to a large extent of an organized type, making use of the available in-house training facilities and supervised by professional instructors, is both personally motivated, as well as formally imposed and duly verified by the employer.

### Metabolic syndrome and pulmonary function

The results of the present study revealed that the MetS subjects had significantly lower FEV1, FVC and VC% predicted than the non-MetS subjects, although the values obtained in both groups were within normal limits. No differences were found as regards FEV1/FVC and FEV1/VC ratio. Rogliani et al. reported the results quite similar to ours, when they compared pulmonary function test results between the non-smoker subjects with and without MetS [84]. Several population-based studies documented that restrictive pulmonary function impairment was associated with an increased risk of MetS [21,24,25,84,85]. Another study revealed, though, that FEV1 was an independent predictor of MetS development [23].

Taking into account that only certain subjects had the values of selected pulmonary function parameters under the lower level of limits, we may well assume that our cohort had normal pulmonary function, i.e. without any impairments. Only ERV% predicted was evidently lower than normal, and the MetS subjects had significantly lower value of this parameter in comparison to the non-MetS subjects. It might well be that the lower value of ERV might have an impact on the lower value of VC% predicted in this group. The results of residual volume and total lung capacity might help resolve this issue.

Lower pulmonary function in the MetS group may result from obesity which characterizes this group. In our study, BMI and waist circumference negatively correlated with several pulmonary function parameters, although by far the strongest relationship was encountered with ERV. Obesity may reduce chest wall compliance, impede diaphragm movement, increase thoracic pressure during expiration, and lead to the closure of peripheral lung units [86]. In a population-based study abdominal obesity was the key determinant of the association between MetS and the pulmonary function impairment [22].

The most likely mechanism that links the impaired pulmonary function with MetS consists in systemic inflammation. Negative associations between CRP levels and several pulmonary function parameters were established. Higher CRP and glucose levels, coronary plaque burden, and lower pulmonary function results in our MetS subjects are corroborated by other studies [23,87–89]. Hsiao et al. found negative correlation between CRP and FEV1 [23]. Systemic inflammation was inversely linked with the quartile of the lowest FVC or FEV1 (% predicted), therefore suggesting its crucial role in the decline of pulmonary function [87]. In the epidemiological study, reduced FEV1 increased the risk of CV mortality irrespective of age, gender and smoking history [88]. Park et al. demonstrated that prevalence of MetS and coronary artery calcification score significantly increased, as FVC or FEV1 values decreased [89]. The results of the Moli-sani Project, pursued on an adult general population, revealed that pulmonary function decline was associated with the estimated risk of CVD in 10 years, as measured by the COURE risk score [90]. The CUORE risk score was inversely associated with FEV1, FVC and total lung capacity only. The authors concluded that a restrictive pattern rather than the airway obstruction was related in particular to a worse cardiovascular risk. Additional research is required to unequivocally determine which type of the pulmonary function abnormalities is associated with cardiovascular risk, as well as what is the potential mechanism that links the impaired pulmonary function with MetS.

## Limitations

Some limitations of our study consist in there being no control groups, and the fact that 65.53% of the subjects only underwent CTCA. It might well be that the effects of stress, as demonstrated in our study, are smaller due to some repressive mechanisms (crowding stress) in this occupational group. The application of the lies scale and self-assessment questionnaire used for the evaluation of the intensity of individual physical activity might well facilitate a more reliable assessment. The fact that we did not perform bodypletysmography was yet another limitation of the present study.

## Conclusions

In conclusion, our cohort study demonstrated high prevalence of MetS and CVD risk factors in police officers. The association of MetS with coronary artery atherosclerosis was weaker than with concomitant, classical CVD risk factors, as detected by CTCA. Stress increased the chance for MetS prevalence, was associated with its components, and also indirectly with the prevalence of coronary plaque. In contrast, leisure-time physical activity reduced the chances of developing MetS. There was neither any relationship between stress and carotid arteries atherosclerosis, nor one with the endothelial function. The lower, though still normal values of the pulmonary function test variables in the MetS subjects may indicate the impact of obesity, or systemic inflammation often associated with this syndrome. This is the first study demonstrating the relationship of MetS and the co-existence of CVD risk factors with prevalence of coronary atherosclerosis, as confirmed by CTCA test in an occupational group exposed to stress. Our findings have application potential both in the clinical practice and public health policy-making. Early primary prevention and a comprehensive therapeutic intervention with regard to CVD risk factors may also have appreciable potential to effectively reduce overall risk of CV events and prevent pulmonary dysfunction in this type of subjects.

## Supporting Information

S1 Table Spearman correlation coefficients between metabolic components, pro-inflammatory markers, FMD, carotid IMT, blood pressure and perceived stress. (DOC)

S2 Table Spearman correlation coefficients between the pulmonary function parameters, BMI, metabolic syndrome components and pro-inflammatory markers. (DOC)
