# Correction: The Relationship of Metabolic Syndrome with Stress, Coronary Heart Disease and Pulmonary Function - An Occupational Cohort-Based Study

**DOI:** 10.1371/journal.pone.0139408

**Published:** 2015-09-24

**Authors:** Miroslaw Janczura, Grazyna Bochenek, Roman Nowobilski, Jerzy Dropinski, Katarzyna Kotula-Horowitz, Bartosz Laskowicz, Andrzej Stanisz, Jacek Lelakowski, Teresa Domagala

Table 1 has been corrected for improved readability. Please see the corrected Table 1 here.

**Table 1:** Clinical characteristics of the study subjects.

Study variable	Value
N (F)	235 (19)
Age, years*	40.97 (6.26)
Framingham Risk Score*	5.82 (5.11)
W/H ratio*	0.94 (0.06)
Overweight, n (%)	101 (42.97)
Obesity, n (%)	103 (43.83)
Dyslipidemia, n (%)	120 (51.06)
Diabetes mellitus, n (%)	7 (2.97)
Hypertension, n (%)	104 (44.25)
Stable CHD, n (%)	17 (7.23)
Positive family history of CHD, n (%)	128 (54.46)
History of myocardial infarction, n (%)	1 (0.42)
PCI, n (%)	5 (2.13)
Smoking status, n (%)	
Never, Current, Past, Pack-years*	96 (40.85), 79 (33.62), 60 (25.53), 17.68 (11.83)
COPD, n (%)	0
Treatments, n (%)	
Aspirin, ACEI/ARB, β-blocker, Ca-blocker, Diuretics, Lipid-lowering drugs, Anti diabetic drugs	8 (3.40), 44 (18.72), 42 (17.87), 14 (5.97), 30 (12.76), 13 (5.53), 7 (2.97)

* Data are expressed as mean (SD); Framingham Risk Score, 10-year risk of developing coronary heart disease; W/H ratio, waist/hip; CHD, coronary heart disease; PCI, percutaneous coronary intervention; BMI, body mass index; ACEI, Angiotensin converting enzyme inhibitors; ARB, Angiotensin II receptor blockers; COPD, chronic obstructive pulmonary disease.

Obesity was defined as BMI>30.0 kg/m^2^ and overweight as BMI >25.0 kg/m^2^.

Table 2 has been corrected for improved readability. Please see the corrected Table 2 here.

**Table 2:** Characteristics of the study subjects stratified by metabolic syndrome status.

Study variable	Metabolic Syndrome	Non-Metabolic Syndrome	p value
N (F)	109 (4)	126 (15)	0.038
Age, years	42.54 (6.75)	39.61 (5.48)	<0.001
Framingham Risk Score	8.32 (5.26)	3.43 (2.99)	<0.0001
BMI, kg/m^2^	32.27 (3.73)	27.78 (3.28)	<0.0001
W/H ratio	0.98 (0.04)	0.91(0.06)	<0.0001
Systolic blood pressure, mm Hg	137.93 (16.93)	124.15 (15.53)	<0.0001
Diastolic blood pressure, mm Hg	87.66 (10.48)	80.36 (9.79)	<0.0001
Stable CHD, n (%)	12 (11.01)	5 (3.96)	0.053
Positive result of stress ECG; n (%)*	18 (18.37)	7 (6.60)	0.012
Coronary plaque burden, n (%)**	29 (37.66)	14 (18.18)	0.007
FMD, %	7.80 (3.23)	8.17 (3.71)	0.59
IMT, mm	0.59 (0.10)	0.54 (0.09)	0.0002
Smoking status, n (%)			
Never, Current, Past	35 (32.11), 40 (36.69), 34 (31.19)	61 (48.41), 39 (30.95), 26 (20.63)	0.01, 0.35, 0.06
Total cholesterol, mmol/L	5.61 (1.01)	5.02 (0.88)	<0.0001
LDL cholesterol, mmol/L	3.25 (0.86)	3.02 (0.76)	0.056
HDL cholesterol, mmol/L	1.12 (0.25)	1.37 (0.35)	<0.0001
Triglycerides, mmol/L	2.92 (1.76)	1.35 (0.75)	<0.0001
Glucose, mmol/L	5.72 (1.29)	5.08 (0.39)	<0.0001
CRP, mg/L	2.18 (2.35)	1.55 (1.78)	0.0001
TNF-α, pg/mL	1.70 (2.85)	1.25 (2.04)	0.06

Data are expressed as mean (SD); BMI, body mass index; W/H, waist/hip; CHD, coronary heart disease; FMD, flow-mediated dilation; IMT, intima-media thickness; LDL, low density lipoprotein; HDL, high density lipoprotein; CRP, C-reactive protein; TNF-α, tissue necrotic factor-α;

* n (%) subjects who underwent stress ECG;

** n (%) subjects who underwent computed tomography coronary angiography.
