# Supplementary material for: The Relationship of Metabolic Syndrome with Stress, Coronary Heart Disease and Pulmonary Function - An Occupational Cohort-Based Study
Source: PLoS One. 2015 Aug 14;10(8):e0133750. doi: 10.1371/journal.pone.0133750 (PMC4537246; doi:10.1371/journal.pone.0133750)
Supplement: S1 Table — (DOC) [file pone.0133750.s001.doc]

S1 Table. Spearman correlation coefficients between metabolic components, pro-inflammatory markers, FMD, carotid IMT, blood pressure and perceived stress.

| **Variable** | **Waist circumference** | **Total cholesterol** | **LDL cholesterol** | **HDL cholesterol** | **Triglycerides** | **Glucose** | **SBP** | **DBP** | **FMD** | **IMT** |
| --- | --- | --- | --- | --- | --- | --- | --- | --- | --- | --- |
| **Age** | 0.12 | 0.16 | 0.06 | -0.03 | 0.23 | 0.25 | 0.22 | 0.20 | -0.16 | 0.34 |
|  | 0.06 | 0.01 | 0.33 | 0.55 | 0.0002 | <0.0001 | 0.0006 | 0.002 | 0.01 | <0.0001 |
| **BMI** | 0.85 | -0.05 | -0.04 | -0.35 | 0.41 | 0.41 | 0.29 | 0.32 | -0.11 | 0.24 |
|  | <0.0001 | 0.44 | 0.49 | <0.0001 | <0.0001 | <0.0001 | <0.0001 | <0.0001 | 0.10 | 0.0001 |
| **CRP** | 0.37 | 0.017 | 0.02 | -0.15 | 0.12 | 0.14 | 0.17 | 0.26 | -0.06 | 0.15 |
|  | <0.0001 | 0.79 | 0.69 | 0.02 | 0.06 | 0.03 | 0.009 | <0.0001 | 0.33 | 0.02 |
| **TNF-α** | 0.30 | -0.013 | -0.67 | -0.18 | 0.20 | 0.08 | 0.06 | 0.01 | -0.07 | 0.12 |
|  | <0.0001 | 0.83 | 0.32 | 0.006 | 0.002 | 0.17 | 0.31 | 0.79 | 0.26 | 0.07 |
| **Fibrinogen** | 0.13 | 0.06 | -0.07 | -0.10 | 0.02 | 0.02 | 0.15 | 0.16 | -0.11 | 0.10 |
|  | 0.04 | 0.29 | 0.29 | 0.10 | 0.77 | 0.75 | 0.02 | 0.01 | 0.10 | 0.12 |
| **FMD** | -0.19 | -0.008 | -0.001 | 0.04 | -0.07 | -0.06 | -0.06 | -0.05 |  | -0.17 |
|  | 0.004 | 0.89 | 0.97 | 0.58 | 0.27 | 0.34 | 0.30 | 0.43 | - | 0.008 |
| **IMT** | 0.22 | 0.14 | 0.09 | -0.20 | 0.24 | 0.18 | 0.18 | 0.17 | -0.17 |  |
|  | 0.0005 | 0.02 | 0.18 | 0.001 | 0.0002 | 0.005 | 0.004 | 0.01 | 0.008 | - |
| **PSS** | 0.17 | 0.11 | 0.09 | -0.04 | 0.19 | 0.03 | 0.19 | 0.17 | 0.08 | 0.15 |
|  | 0.03 | 0.14 | 0.23 | 0.56 | 0.002 | 0.69 | 0.01 | 0.03 | 0.26 | 0.052 |

Data are presented as r (upper line) and p (lower line)

BMI, body mass index; LDL, low density lipoprotein; HDL, high density lipoprotein; CRP, C-reactive protein; TNF-α, tissue necrotic factor-α; FMD, flow-mediated dilation; IMT, intima-media thickness; SBP, systolic blood pressure; DBP, diastolic blood pressure; PSS, perceived stress score.
