# Supplementary material for: The Relationship of Metabolic Syndrome with Stress, Coronary Heart Disease and Pulmonary Function - An Occupational Cohort-Based Study
Source: PLoS One. 2015 Aug 14;10(8):e0133750. doi: 10.1371/journal.pone.0133750 (PMC4537246; doi:10.1371/journal.pone.0133750)
Supplement: S2 Table — (DOC) [file pone.0133750.s002.doc]

S2 Table. Spearman correlation coefficients between the spirometry parameters, BMI, metabolic syndrome components and pro-inflammatory markers.

| **Variable** | **Waist circumference** | **BMI** | **HDL cholesterol** | **Triglycerides** | **Glucose** | **SBP** | **DBP** | **CRP** | **TNF-α** |
| --- | --- | --- | --- | --- | --- | --- | --- | --- | --- |
| **FEV_1_** | -0.18 | -0.11 | -0.03 | -0.15 | -0.11 | -0.14 | -0.09 | -0.16 | 0.03 |
|  | 0.01 | 0.11 | 0.64 | 0.03 | 0.11 | 0.05 | 0.19 | 0.03 | 0.68 |
| **VC** | -0.24 | -0.17 | 0.03 | -0.24 | -0.17 | -0.17 | -0.13 | -0.13 | 0.02 |
|  | 0.0007 | 0.01 | 0.61 | 0.0008 | 0.01 | 0.01 | 0.06 | 0.08 | 0.70 |
| **FVC** | -0.26 | -0.20 | 0.09 | -0.25 | -0.18 | -0.15 | -0.11 | -0.20 | 0.03 |
|  | 0.0003 | 0.004 | 0.19 | 0.0003 | 0.01 | 0.03 | 0.10 | 0.008 | 0.67 |
| **FEV1%VC** | -0.004 | -0.002 | -0.01 | 0.03 | -0.02 | 0.07 | 0.03 | -0.03 | 0.02 |
|  | 0.95 | 0.96 | 0.80 | 0.62 | 0.68 | 0.32 | 0.68 | 0.66 | 0.74 |
| **FEV1%FVC** | -0.03 | 0.02 | -0.07 | 0.01 | -0.05 | -0.004 | -0.01 | 0.03 | -0.03 |
|  | 0.60 | 0.76 | 0.33 | 0.81 | 0.49 | 0.95 | 0.89 | 0.65 | 0.68 |
| **FEF25** | -0.07 | -0.02 | -0.05 | -0.08 | -0.12 | -0.009 | -0.008 | -0.008 | 0.05 |
|  | 0.29 | 0.71 | 0.48 | 0.23 | 0.09 | 0.89 | 0.90 | 0.91 | 0.43 |
| **FEF50** | -0.007 | 0.06 | -0.10 | -0.01 | -0.07 | -0.01 | -0.02 | 0.03 | 0.04 |
|  | 0.92 | 0.39 | 0.17 | 0.84 | 0.31 | 0.86 | 0.72 | 0.65 | 0.52 |
| **FEF75** | -0.11 | -0.06 | -0.08 | -0.03 | -0.10 | -0.05 | -0.06 | -0.21 | 0.02 |
|  | 0.11 | 0.36 | 0.25 | 0.63 | 0.15 | 0.49 | 0.35 | 0.005 | 0.73 |
| **ERV** | -0.34 | -0.36 | 0.10 | -0.24 | -0.21 | -0.17 | -0.17 | -0.21 | -0.01 |
|  | <0.0001 | <0.0001 | 0.17 | 0.0009 | 0.04 | 0.02 | 0.02 | 0.006 | 0.82 |

Data are presented as r (upper line) and p (lower line)

BMI, body mass index; LDL, HDL, high density lipoprotein; CRP, C-reactive protein; TNF-α, tissue necrotic factor-α; SBP, systolic blood pressure; DBP, diastolic blood pressure; FEV_1_, forced expiratory volume in 1 second; VC, Vital Capacity; FVC, forced vital capacity; FEV_1_%VC, Tiffenau index; FEV1/FVC,

a ratio of forced expiratory volume in 1 second (FEV1) to a forced vital capacity (FVC); FEF, forced expiratory flow; ERV, expiratory reserve volume.
